# Lactophenin

**Published:** 1897-05

**Authors:** J. A. Childs

**Affiliations:** Atlanta, Ga.


### LACTOPHENIN.

By J. A. CHILDS, M.D.,

Atlanta, Ga.

Lactophenin is a phenacetin in which the acetic acid is replaced by lactic acid. It consists of colorless, odorless crystals, with a slightly bitter taste, which melts at 117° to 118° C., and are soluble in about 500 parts of cold water or in about nine parts of alcohol.

Lactophenin is an antipyretic and an analgesic drug that is of decided value in fevers, especially typhoid. The great value claimed for it over other antipyretics is its freedom from depressing after-effects, which we frequently see in the coal-tar derivatives when given in large doses.

The excellent results obtained from this remedy in acute articular rheumatism demands its trial in this painful and dangerous disease. That lactophenin is less liable to affect the heart than many other antipyretics is shown in the large doses that can be given with good results without any harmful effects. The antipyretic dose is ten grains and the hypnotic twelve or fifteen grains. With the introduction of the coal-tar derivatives there was placed in the hands of the physician valuable drugs for the alleviation of pain and the reduction of high temperatures. That these preparations have in a measure lessened the use of opium and its alkaloids there can be no doubt, and if they only possessed analgesic properties they would be valuable additions to our pharmacopœia. While water is the ideal antipyretic agent in long continued fevers or pneumonia where the heart's action is weakened (and in many cases it would be the best means to reduce the temperature where the heart's action is not weakened), still this method is contra-indicated in many cases where the patient is excitable or the inconvenience of its use. That many lives were shortened by the indiscriminate use, in inexperienced hands, of the coal-tar preparations when first introduced there can be no doubt, but a more thorough knowledge of their toxic effect have minimized these results to some extent. What we have needed in the past is a drug like lactophenin, that reduces temperature, relieves pain, quiets the nervous symptoms, and does not produce the depressed or collapsed condition that we frequently see in other drugs.

Professor Stein (*Allgem. Med. Centr. Ztg.*) says of lactophenin: "The great advantage of the drug consists in the absence of shivering fits on the subsequent rise of temperature." Although some authors have considered this a never-failing property, Stein says that he observed shivering fits in eight cases, but only after the temperature had been suddenly reduced by large doses to considerable degree.

Stein warmly recommends the employment of lactophenin in typhus abdominalis, especially if hydro-therapeutic treatment is not possible nor advisable whilst the reduction of temperature is absolutely necessary. He does not attribute a specific action to lactophenin in typhus, but he was able to control the temperature during the whole course of the disease, to the great comfort of the patient. As an algesic remedy, Stein also obtained exceptionally good results with large doses—up to 120 grains per day—in articular rheumatism, and he also employed it as a sedative in headaches of various origin. Lactophenin exerted no appreciable influence upon the strength and frequency of the pulse. He comes to the conclusion that "the indications for the employment of lactophenin are substantially the same as for phenacetin, but it is distinctly preferable to the latter on account of its relative freedom from toxic action."

*Pediatrics* (March 15, 1897) quotes from a "Report on New Remedies," by Dr. P. Phillip: "Strauss, Schlutius and Riede publish their experience with lactophenin. Its sedative qualities, which make it useful, particularly in typhoid fever, are praised by most of its friends. According to Stein, it may be used wherever phenacetin is indicated. Because of its relative safety and possibility of longer use it is much superior to phenacetin. Riede calls it a specific in acute articular rheumatism, producing no ear-symptoms or depression of the heart, as does salicylic acid and salophen."

Dr. Brodnax says (in *Medical Summary*, Feb., 1897), it is "a pleasant pain reliever. The dose is small, no cyanosis has been noted as yet, and the restful sleep that follows five grains (for adults), on retiring, is as near natural sleep as possible. So far as I have experimented on myself and others, I find no increase of the dose necessary to produce the desired result. Where the continued use is wanted, smaller doses will answer, say two grains every hour or two."

Dr. Beverly Drake Harrison, in a paper before the Upper Peninsular (Mich.) Medical Association on "Treatment of Typhoid Fever" (*Physician and Surgeon*, Nov., 1896), basing his methods on experience with 500 cases with only two deaths, recommends lactophenin "to eliminate toxins and to promote skin drainage"; he says it has a calming and hypnotic effect, no influence on the heart, and breathing remains unaffected, while the temperature is reduced gradually. His testimony is thus summarized: "I cannot speak too highly of lactophenin as an antipyretic and hypnotic. I have used it altogether in my practice during the past three years to the exclusion of all other antipyretics, and I have yet to learn of a single case in which it had the slightest depressing effect upon the heart or circulation."

Dr. W. C. Buckley, of Philadelphia, says (in *The Laryngoscope*, Feb., 1897): Lactophenin reduces abnormal temperature, but does not seem to exert any marked influence upon the circulation or respiration. I have used it in pneumonia, influenza, scarlatina, acute tuberculosis accompanied by fever, and septicemia, with excellent results. In the high temperature and restlessness of enteric fever (typhoid) it has also served me a most excellent purpose; here a child may take one or two grains with pleasant effect. The full adult dose is from four to sixteen grains. In giving this remedy the proper plan is to begin with small doses, and increase according to the effect produced."

---

### BRONCHIAL ASTHMA.

℞ Sodii iodidi.................................................ʒ i.<br>

Ammonii carbonat.........................................ʒ i.<br>

Tr. lobelia...................................................ʒ ii.<br>

Spts. chloroformi..........................................ʒ iv.<br>

Vini ipecac....................................................ʒ i.<br>

Infus. senegæ, q. s. ad...................................℥ vi.<br>

M. Tablespoonful in wineglassful of water every three or four hours.

—DR. RAINEAR (*Codex Med.*).

